# Fatigue and Recovery Time Course After Female Soccer Matches: A Systematic Review And Meta-analysis

**DOI:** 10.1186/s40798-022-00466-3

**Published:** 2022-06-03

**Authors:** Karine Naves Oliveira Goulart, Cândido Celso Coimbra, Helton Oliveira Campos, Lucas Rios Drummond, Pedro Henrique Madureira Ogando, Georgia Brown, Bruno Pena Couto, Rob Duffield, Samuel Penna Wanner

**Affiliations:** 1 Exercise Physiology Laboratory, School of Physical Education, Physiotherapy and Occupational Therapy, Universidade Federal de Minas Gerais, Av. Antônio Carlos, 6627. Pampulha, Belo Horizonte, MG 31270-901 Brazil; 2 School of Sport, Exercise and Rehabilitation, Faculty of Health, University of Technology Sydney (UTS), Moore Park, NSW Australia; 3 Department of Physiology and Biophysics, Institute of Biological Sciences, Universidade Federal de Minas Gerais, Belo Horizonte, MG Brazil; 4 Department of Biological Sciences, Universidade do Estado de Minas Gerais – Unidade Carangola, Carangola, MG Brazil; 5 School of Health and Behavioural Sciences, University of the Sunshine Coast, Sippy Downs, QLD Australia; 6 Medical Department, Sydney, Football Australia Australia

**Keywords:** Creatine kinase, Countermovement jump, Cortisol, Estradiol, Female soccer, Muscle soreness, Performance, Recovery, Women

## Abstract

**Background:**

This study aimed to analyze the extent of fatigue responses after female soccer matches and the ensuing recovery time course of performance, physiological, and perceptual responses.

**Methods:**

Three databases (PubMed, Web of Science, and SPORTDiscus) were searched in October 2020 and updated in November 2021. Studies were included when participants were female soccer players, regardless of their ability level. Further, the intervention was an official soccer match with performance, physiological, or perceptual parameters collected pre- and post-match (immediately, 12 h, 24 h, 48 h, or 72 h-post).

**Results:**

A total of 26 studies (*n* = 465 players) were included for meta-analysis. Most performance parameters showed some immediate post-match reduction (effect size [ES] = − 0.72 to − 1.80), apart from countermovement jump (CMJ; ES = − 0.04). Reduced CMJ performance occurred at 12 h (ES = − 0.38) and 24 h (ES = − 0.42) and sprint at 48 h post-match (ES = − 0.75). Inflammatory and immunological parameters responded acutely with moderate-to-large increases (ES = 0.58–2.75) immediately post-match. Creatine kinase and lactate dehydrogenase alterations persisted at 72 h post-match (ES = 3.79 and 7.46, respectively). Small-to-moderate effects were observed for increased cortisol (ES = 0.75) and reduced testosterone/cortisol ratio (ES = -0.47) immediately post-match, while negligible to small effects existed for testosterone (ES = 0.14) and estradiol (ES = 0.34). Large effects were observed for perceptual variables, with increased fatigue (ES = 1.79) and reduced vigor (ES = − 0.97) at 12 h post-match, while muscle soreness was increased immediately post (ES = 1.63) and at 24 h post-match (ES = 1.00).

**Conclusions:**

Acute fatigue exists following female soccer matches, and the performance, physiological, and perceptual parameters showed distinctive recovery timelines. Importantly, physical performance was recovered at 72 h post-match, whereas muscle damage markers were still increased at this time point. These timelines should be considered when planning training and match schedules. However, some caution should be advised given the small number of studies available on this population.

**Registration:**

The protocol for this systematic review was pre-registered on the International Prospective Register of Systematic Reviews (PROSPERO, Registration Number: CRD42021237857).

**Supplementary Information:**

The online version contains supplementary material available at 10.1186/s40798-022-00466-3.

## Key Points

- Physical performance parameters were impaired until 48 h post-match in female soccer players.
- Parameter-specific responses were observed for inflammatory and muscle damage markers, with IL-6 and TNF-α altered only immediately post-match, C-reactive protein until 48 h, while creatine kinase and lactate dehydrogenase remained increased at 72 h post-match.
- Limited data for extended recovery time courses exist in female soccer, especially for perceptual parameters.

## Background

Soccer players encounter high physical, physiological, and psychological match loads that can result in both temporary (acute) and ongoing (residual) fatigue [1–5]. These match-based responses affect ensuing training and match preparation and are highly researched in male populations [6–8]. Despite the popularity of female soccer (including growing financial support and participation rates [9–12]), limited research exists on women’s compared to men’s soccer. However, publications on women’s soccer per year are progressively increasing from 1939 (one study) to 2019 (202 studies) and tend to peak around the FIFA World Cup years [13]. Within this publication growth, the most frequently investigated areas relate to sports medicine (injuries), while the fatigue and recovery responses to acute and chronic bouts of soccer occupied the fourth position [13], which shows the fledgling, but growing, focus of research in this area.

The recovery process, which follows physically, emotionally, and cognitively demanding activities, underpins the return of players to match or training readiness, particularly in contexts of congested match and training schedules [14]. Soccer matches result in considerable physical demands and both physiological (systemic and neuromuscular fatigue and damage) and cognitive (decision making) loads [15, 16]. For instance, physical demands include total distances between 9 and 11 km, with ≈1.5 km in high-intensity running, alongside ≈1400 activity changes every 3–6 s [17–20]. These demands result in substantial physical and perceptual fatigue, which requires consideration when planning training or match readiness. Given the diverse effects of match loads, recovery is of a multi-factorial nature, with performance, physiological, and perceptual states showing heterogeneous post-match recovery time course [21]. Understanding the recovery process with a multi-factorial view can support subsequent training prescription and recovery protocols, whilst also ensuring evidence-based practices are used for determining a minimum interval between matches.

A previous systematic review and meta-analysis of match-related fatigue in soccer reported physical performance, physiological, and perceptual responses remain affected until ≈72 h post-match [6]. However, from the 42 studies included in this meta-analysis, only 10 reported post-match recovery in females. Thus, although male and female outcomes have been analyzed collectively, the findings were possibly more influenced by male data. Considering that sex-based differences exist for match-induced fatigue due to different match locomotor loads and activity patterns [22], muscle damage, and inflammation responses [2], the post-match recovery in female soccer merits investigation. For example, sex differences in power- and endurance-related physical capacities of soccer players exist, with women presenting lower values in sprints, jumps, and intermittent endurance [23]. Further, post-match fatigue is reduced in male players with higher physical qualities (i.e., high-intensity running ability and lower body strength) [24].

When interpreting post-match fatigue and recovery responses, it is important to acknowledge the match loads encountered. Although historical research suggests females cover less distance in matches [22], the most recent men’s (Russia 2018) and women’s (France 2019) FIFA World Cups showed the total distances covered were similar, albeit the average female players cover less distance at higher speeds [25, 26]. Thus, substantial differences between men’s and women’s locomotor match loads seem to have been reduced during the last decade. Investigation in female players is also important considering that menstrual cycle effects on performance-related parameters in elite athletes remain inconclusive [27]; however, recent literature suggests there could be potential effects of the menstrual cycle phase on recovery [28] and wellness [29]. Therefore, it is unclear whether applying information on men’s post-match recovery to women would provide an accurate understanding of female responses, which is essential to plan training and recovery for female players adequately. The current study aimed to analyze the acute and residual fatigue after female soccer matches and the recovery time course of performance, physiological, and perceptual responses. The research question was defined using the PICO model. Population: Female soccer players. Intervention: Soccer matches. Comparators: Changes between pre- and post-match (i.e., immediately post, 12, 24, 48, and 72 h post-match). Outcomes: Physical performance, physiological parameters, and perceptual responses.

## Methods

The protocol for this systematic review was pre-registered on the International Prospective Register of Systematic Reviews (PROSPERO, registration number: CRD42021237857). The systematic review and meta-analysis was conducted according to the Preferred Reporting Items for Systematic Reviews and Meta-Analysis (PRISMA) guidelines [30].

### Search Strategy

Studies were searched for in three databases: PubMed (MEDLINE), Web of Science, and SPORTDiscus (EBSCO). The following search terms were combined using Boolean operators: (female soccer OR women soccer OR female football OR women football) AND (match OR game) AND (agility OR change of direction OR delayed onset muscle soreness OR fatigue OR hormones OR immunology OR inflammation OR jump OR menstrual cycle OR mood OR muscle damage OR muscle soreness OR neuromuscular OR performance OR recovery OR repeated sprint OR sprint).

### Study Inclusion and Exclusion Criteria

The following inclusion criteria were set: (1) Participants should be female soccer athletes, regardless of their ability level; (2) intervention should be an official soccer match or a friendly match following official rules; (3) performance parameters screened consisted of vertical jumping tests, speed, agility, strength, change of direction, endurance, and intermittent endurance tests; (4) physiological parameters screened were creatine kinase (CK), lactate dehydrogenase (LDH), C-reactive protein (CRP), cytokines (IL-6 and TNF-α), neutrophils, leukocytes, lymphocytes, cortisol, testosterone, testosterone/cortisol ratio, and estradiol; (5) perceptual parameters included were delayed onset muscle soreness (DOMS), fatigue, and vigor; and (6) all parameters must have been measured at pre- and at some time point post-match.

The search was conducted in October 2020, with no date restrictions, and all included studies were written in English. An update search was conducted in November 2021. Reviews, summaries, and letters were not included, though consulted before exclusion during this first screening phase. Further, the established exclusion criteria were (1) outcome merged with male results or other sports; (2) small-sided games or simulated matches; and (3) female players under 15 years old when matches were less than 90-min length.

### Study Selection and Quality Assessment

**Fig. 1:**
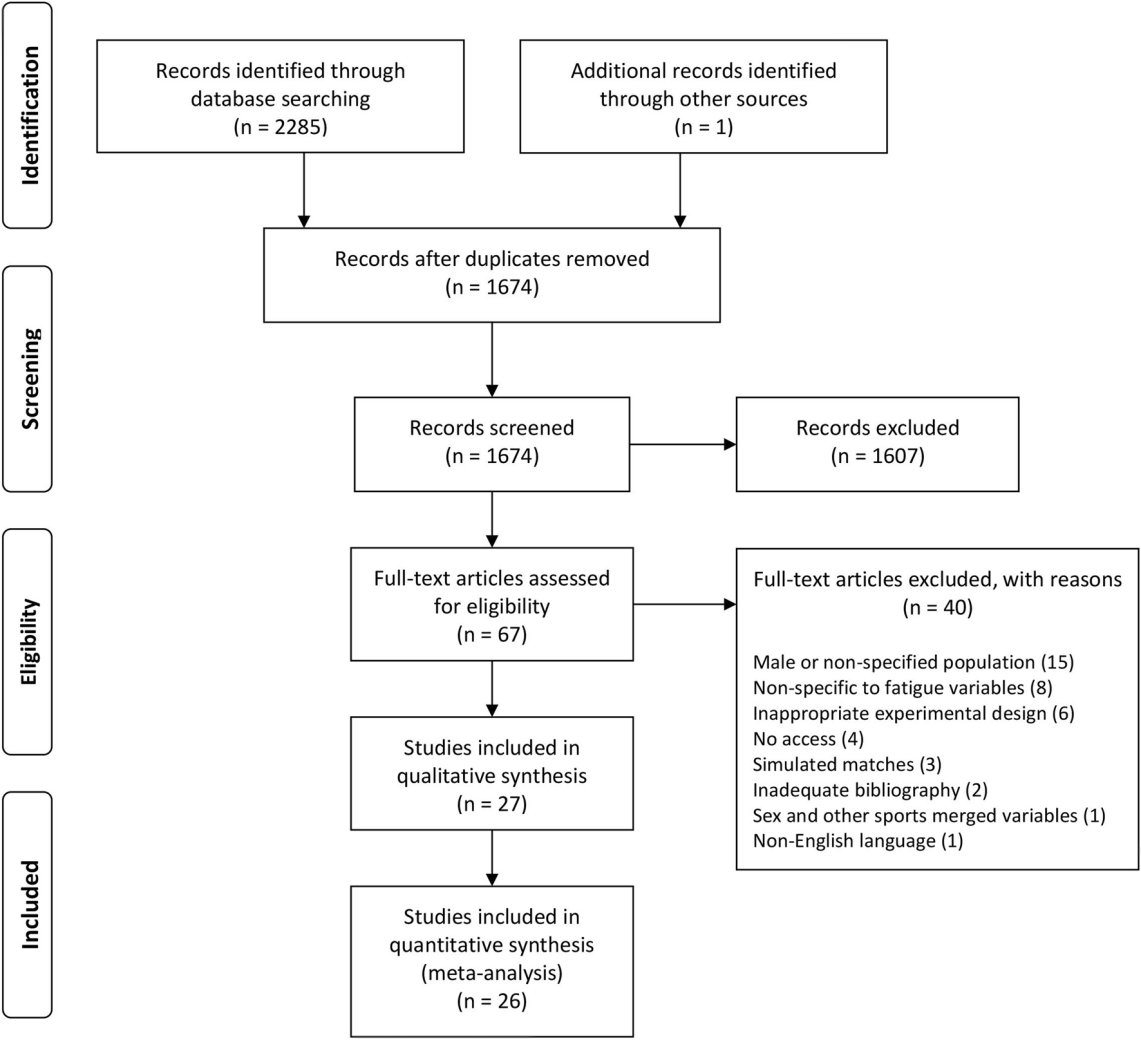
Flow chart of study inclusion process

Studies were searched for and inserted in the Rayyan web application (https://rayyan.qcri.org). Firstly, studies were screened for inclusion, reviewing titles and abstracts by three researchers (KG, HC, PO), with all cases of disagreement discussed until consensus was reached. Inclusion and exclusion decisions were duly labeled in the Rayyan web application [31]. In a second stage, four researchers (KG, HC, PO, and LD) reviewed the remaining full-text manuscripts for exclusion. The exclusion reasons were presented (Fig. 1) and jointly discussed with CC and SW until consensus was reached.

The methodological quality and risk of bias from the selected studies were determined using a qualitative assessment tool, consisting of 13 questions adapted from Silva et al. [6]. This was an independently paired process, with answer categories of “yes,” “partially,” and “no,” scoring 2, 1, or 0, respectively. When the total score difference of a given study was higher than 2, a third researcher completed the qualitative assessment, and the mean score of the three analyses was reported.

### Data Extraction Strategy

Two researchers (KG and HC) independently extracted and included the data in a standardized spreadsheet, according to the type of parameter (i.e., performance, perceptual, or physiological). Performance parameters were analyzed into three subgroups: countermovement jump (CMJ), sprint tests (10, 20, and 30 m), and YoYo intermittent endurance tests level 1 (YoYoIE1) and level 2 (YoYoIE2). Further, data for power and strength-related capabilities (peak torque flexion and extension, maximum voluntary isometric contraction (MVIC) during knee flexion and extension, maximal rate of force development, squat jump peak power and peak force, heel-rise test) and agility tests were included in the table of performance parameters, though not used for meta-analyses due to small sample size. Physiological parameters, measured through blood (plasma or serum), and salivary samples, were divided into three categories: (1) muscle damage markers, (2) inflammatory markers, and (3) mediators of neuroendocrine regulation. Perceptual parameters consisted of Likert scale ratings of delayed onset muscle soreness (DOMS), fatigue, and vigor.

The following information was extracted: first author and year of publication, screened parameter, and measurement time points. Only data from control groups were extracted from intervention studies [32]. Post-match measurements were adjusted to 5 categories according to their respective time point ranges: immediately post-match, 12 h (1–12 h); 24 h (13–24 h); 48 h (25–48 h); and 72 h (49–72 h) post-match. Data were extracted from graphs using WebPlotDigitizer (v4.4, Pacifica, CA, USA). When necessary, corresponding authors were contacted by email. Further, study characteristics, including match conditions and subject characteristics, are shown in Table 1.

**Table 1:** Description of the studies included in the systematic review

	Anthropometric characteristics						Match conditions			
Study	N	Age (years)	Height (cm)	Body mass (kg)	Body fat (%)	VO_2max_ (ml.kg^−1^.min^−1^)	Level	Period	Time playing (min)	Environment
Ai Ishida et al. [33]	12	20.7 ± 2.3	164.5 ± 6.0	64.4 ± 7.2						
Andersen et al. [34]	27	Denmark: 22 ± 2 Norway: 21 ± 6	Denmark: 169 ± 7 Norway: 170 ± 7	Denmark: 61 ± 7 Norway: 62 ± 7			HL		90	
Andersson et al. [1]	17	Active recovery: 22.6 ± 4.2 Passive recovery: 21.6 ± 2.6	Active recovery: 167.1 ± 5.7 Passive recovery: 167.2 ± 4.7	Active recovery: 63.3 ± 7.1 Passive recovery: 65.0 ± 4.6		Active recovery: 55.4 ± 3.6 Passive recovery: 53.8 ± 2.3	E		90	12 °C, light rain
Andersson et al. [35]	10	23 ± 13	167 ± 19	64 ± 19		54 ± 3	E	Mid-season	90	12 °C, light rain
Bonilla et al. [36]	14	27.8 ± 5.0	176.1 ± 4.1	71.6 ± 3.3	18.5 ± 3.5		HL			
Broodryk et al. [37]	8	23.1 ± 3.2	158.9 ± 6.4	54.7 ± 4.2			C		90	23 °C
Casanova et al. [3]	18	23.1 ± 4.3	169.4 ± 5.4	58.2 ± 4.3			E	Early season	Minimum 10	
Casanova et al. [38]	20	22.9 ± 4.2	170.2 ± 5.7	59.3 ± 4.6	18.7 ± 3.1		E	Early season		
Casto et al. [39]	25	18–22					C			
Casto et al. [40]	25	18–22					C			
Edwards [41]	18	18–22					C	End of season		
Goulart et al. [32]	10	25.1 ± 5.9	162.0 ± 6.0	58.9 ± 6.2	17.9 ± 3.3	48.8 ± 0.4	E	Regular season	84.0 ± 14.5	33 ± 5 °C, 39 ± 13% RH
Gravina et al. [42]	28	elite: 25 ± 5 sub-elite: 18.3 ± 1.5		elite: 61.0 ± 7.4 sub-elite: 61.9 ± 9.8	elite: 15.5 ± 2.9 sub-elite: 18.4 ± 3.3		E/HL			
Haneishi et al. [43]	20	starters: 20.2 ± 2.0 nonstarters: 20.5 ± 1.7	starters: 166 ± 7.7 nonstarters: 166 ± 4.6	starters: 58.3 ± 7.3 nonstarters: 64.6 ± 5.8	starters: 16.6 ± 4.8 nonstarters: 19.1 ± 2.5		C	Regular season		21 °C, 30% RH
Hassmen and Blomstrand [44]	9	22.1 ± 3.6					E	Regular season		
Hoffman et al. [45]	starters: 9 nonstarters: 10	starters: 20.0 ± 1.0 nonstarters: 18.2 ± 0.4	starters: 162.4 ± 3.8 nonstarters: 164.2 ± 7.8	starters: 57.7 ± 4.7 nonstarters: 62.1 ± 8.3			HL	End of season	starters: 56.5 ± 14.0 nonstarters: 29.0 ± 13.9	7 °C, 30% RH
Hughes et al. [46]	U17: 12	U17: 15.8 ± 0.3	U17: 166.8 ± 3.4	U17: 58.6 ± 4.7			E	Regular season	U17: 90	7 °C, 72% RH
Krustrup et al. [47]	14	23 (18–29)	169 (159–180)	60.1 (53.3–69.5)	18.5 (12.7–27.6)	52.3 ± 1.3	E	Regular season		
Maya et al. [5]	16	22.5 ± 2.1	163 ± 7	59.5 ± 6.3			E	End of season	90	
Oliveira et al. [48]	33	24.2 ± 4.8					E	End of season	90	
Pavin et al. [49]	20	20.6 ± 3.9	164 ± 4	59.6 ± 11.6			R		90	29 °C, 45% RH
Póvoas et al. [50]	high-rank players = 6 low-rank players = 7	26 ± 4	170 ± 4	63.4 ± 4.8			E	Pre-season (high-rank team players) Mid-season (low-rank team players)	77 ± 15	21–22 °C, 50–70% RH
Scott et al. [51]	CB: 8 FB: 6 CM: 15 WM: 3 F: 4	CB: 25.3 ± 3.2 FB: 24.1 ± 3.6 CM: 24.5 ± 4.1 WM: 23.2 ± 5.2 F: 25.1 ± 4.9	CB: 170.5 ± 7.1 FB: 168.5 ± 6.2 CM: 167.5 ± 5.3 WM: 166.0 ± 7.1 F: 168.7 ± 5.2	CB: 66.0 ± 5.1 FB: 62.1 ± 4.2 CM: 62.3 ± 4.1 WM: 61.6 ± 5.1 F: 62.7 ± 6.1			E		90	
Snyder et al. [4]	8		168.3 ± 5.6	63.5 ± 6.3			E		77.5 ± 8.9	
Souglis et al. [52]	21	22.9 ± 2.4	168 ± 3	61.0 ± 3.3	15.2 ± 1.0	52.0 ± 1.8	E	Regular season		17–20 °C, 40–50% RH
Souglis et al. [2]	DEF: 10 MID: 10 ATT: 10	DEF: 25.3 ± 3.2 MID: 24.7 ± 3.9 ATT: 23.9 ± 3.0	DEF: 170 ± 3 MID: 168 ± 3 ATT: 170 ± 3	DEF: 60.0 ± 1.4 MID: 58.3 ± 2.5 ATT: 59.4 ± 3.6	DEF: 19.6 ± 1.0 MID: 18.2 ± 0.7 ATT: 19.1 ± 1.0	DEF: 52.1 ± 1.9 MID: 55.4 ± 1.6 ATT: 53.2 ± 2.6	E	Regular season		14–20 °C, 40–60% RH
Tsubakihara et al. [53]	18		161.2 ± 4.1	56.2 ± 5.1	22.2 ± 6.3		C		90	

A= amateur; ATT= attackers; C= college; CB= center back; CM= center midfield; DEF= defenders; E= elite; F= forward; FB= full back; HL= high level; MID= midfielders; R= recreational; RH= relative humidity; and WM= wide midfield. Values are as mean ± standard deviation or as mean (range)

### Statistical Analyses

The mean and standard deviation values of the parameters were obtained from the data provided in the consulted research papers or from the authors’ response upon request (when data extraction was not possible). Heterogeneity was evaluated using the *χ*^2^ test for homogeneity and I^2^ statistic. The effect size (Hedges’ g, if *n* < 10 trials, or Cohen’s d) was calculated for each trial. A weighted-mean estimate of the effect size (ES) was calculated to account for differences in the sample sizes, along with the mean unweighted ES and associated 95% confidence interval (CI). When CI included zero, ES was considered not significant [54, 55]. Threshold values for ES were defined as negligible (< 0.2); small (0.20–0.49); moderate (0.50–0.79); and large (> 0.8) [56]. Meta-analysis was conducted using the Stata^®^ software (v.11.1, College Station, TX, USA) and GraphPad^®^ software (Prism 5.0, San Diego, CA, USA). Measures of statistical heterogeneity for each parameter included in the meta-analysis are provided in the Additional file 1: Supplementary Table 1.

## Results

### Review Statistics

The flow chart showing the study selection process is presented in Fig. 1. A total of 1275, 577, and 433 manuscripts were identified in the Web of Science, PubMed, and SPORTDiscus databases, respectively. Rayyan identified 612 duplicates that were confirmed and removed by the first author (KG). After screening and eligibility phases, 27 studies were included for qualitative analysis, comprising a sample size of 501 female soccer players. Data from Scott et al. [51] were not included in the meta-analyses since fatigue and soreness were reported as estimated marginal means.

### Study Characteristics

The characteristics of the female soccer players and match conditions are summarized in Table 1. A total of 501 female soccer players were included from the reported studies. The playing level was classified as recreational (R), college (C), high level (HL), or elite (E), according to Okholm Kryger et al. [13]. Of note, elite consisted of professional athletes; high level consisted of semiprofessional, sub-elite, high performance, second or third best division athletes; college consisted of players competing for a university or college; and recreational consisted of any amateur but competitive level. The period when matches took place was classified as the early season (group stage), mid-season, end of the season (last week, last game of the soccer season or final match), and regular season (throughout the competition). Further, 15/27 studies mentioned that goalkeepers were not included for analyses, though 12 studies did not specify the playing positions.

## Meta-analyses

In total, 26 studies (*n* = 465 female soccer players) were included in the meta-analyses. The mean and standard deviation of each performance, physiological, and perceptual parameter included in the meta-analyses are presented in Tables 2, 3, 4. We considered a parameter to be recovered when a significant difference from pre-match values was no longer observed in the meta-analysis, thus indicating the absence of acute or residual fatigue.

**Table 2:** Fatigue and recovery time course of performance parameters

Performance parameters							
Study	Variable	Pre	Post	1–12 h	13–24 h	25–48 h	49–72 h
Ai Ishida et al. [33]	CMJ height (cm)	25.4 ± 4.7		22.3 ± 4.5*		24.9 ± 4.6	
	CMJ peak force (N)	739.6 ± 92.8		714.0 ± 66.8		718.2 ± 73.4	
	CMJ peak power (W)	2666 ± 154		2541 ± 156*		2632 ± 165	
Andersen et al. [34]	YoYoIE1 (m)	1480 ± 396	1025 ± 337*				
Andersson et al. [1]	20 m sprint (s)						
	Match 1—Active group	3.18 ± 0.12	3.26 ± 0.12*	Missing	Missing	Missing	3.17 ± 0.12
	Match 1—Passive group	3.17 ± 0.12	3.28 ± 0.16*	Missing	Missing	Missing	3.15 ± 0.16
	Match 2—Active group	3.17 ± 0.12	3.25 ± 0.08				
	Match 2—Passive group	3.15 ± 0.16	3.23 ± 0.12				
	CMJ height (cm)						
	Match 1—Active group	30.5 ± 3.4	29.1 ± 2.9*	29.4 ± 2.9	28.9 ± 2.5	29.2 ± 3.4	29.2 ± 3.1*
	Match 1—Passive group	29.8 ± 3.7	28.4 ± 3.0*	29.9 ± 2.9	29.3 ± 3.2	29.1 ± 3.6	28.9 ± 3.6*
	Match 2—Active group	29.2 ± 3.1	28.6 ± 3.2				
	Match 2—Passive group	28.9 ± 3.6	28.4 ± 3.8				
	Peak torque flexion (°. s^−1^)						
	Match 1—Active group	102 ± 21	93 ± 25*	Missing	Missing	Missing	101 ± 21
	Match 1—Passive group	104 ± 21	95 ± 21*	Missing	Missing	Missing	104 ± 21
	Match 2—Active group	101 ± 21	96 ± 11				
	Match 2—Passive group	104 ± 21	98 ± 12				
	Peak torque extension (°. s^−1^)						
	Match 1—Active group	175 ± 21	165 ± 25*	Missing	Missing	Missing	170 ± 25
	Match 1—Passive group	167 ± 16	154 ± 21*	Missing	Missing	Missing	160 ± 25
	Match 2—Active group	170 ± 25	166 ± 14				
	Match 2—Passive group	160 ± 25	154 ± 21				
Bonilla et al. [36]	CMJ height (cm)	24.7 ± 3.2	24.9 ± 3.9		23.4 ± 4.0*		
	CMJ peak power (W)	2139 ± 347	2154 ± 334		2059 ± 349		
Goulart et al. [32]	CMJ (cm)	29.54 ± 2.56			28.01 ± 1.56#	28.93 ± 1.89	29.81 ± 1.55
	20 m sprint (s)	3.34 ± 0.07			3.466 ± 0.12#	3.422 ± 0.09#	3.366 ± 0.07#
	10 m sprint (s)	1.94 ± 0.03			1.979 ± 0.075	1.968 ± 0.070	1.941 ± 0.043
Hoffman et al. [45]	SJ peak power (W)						
	Starters	3144 ± 656	3167 ± 502		2754 ± 776		
	Nonstarters	3453 ± 546	3559 ± 423		3398 ± 679		
	CMJ peak power (W)						
	Starters	3087 ± 438	2931 ± 477		2608 ± 411*		
	Nonstarters	3352 ± 593	3433 ± 604		3229 ± 656		
	SJ peak force (N)						
	Starters	1014 ± 115	1016 ± 80		919 ± 151		
	Nonstarters	1178 ± 276	1141 ± 171		1142 ± 182		
	CMJ peak force (N)						
	Starters	923 ± 109	929 ± 128		836 ± 128		
	Nonstarters	1097 ± 174	1111 ± 275		1047 ± 216		
	Maximal rate of force development (kg × 10^3.^ s^−1^)						
	Starters	17.0 ± 3.7	17.8 ± 4.3		16.5 ± 3.6		
	Nonstarters	17.4 ± 3.3	17.4 ± 2.9		19.1 ± 3.4		
Krustrup et al. [47]	CMJ height (cm)	35 ± 4	36 ± 4				
	30 m sprint (s)	4.86 ± 0.22	5.06 ± 0.22*				
	YoYo IE2 (m)	1265 ± 498	484 ± 187*				
Pavin et al. [49]	T test agility (s)	11.8 ± 0.5	12.8 ± 0.5*				
	Heel-rise test (rep)	35.4 ± 2.9	24.0 ± 4.0*				
	YoYoIE2 (m)	711.3 ± 93.1	496.0 ± 96.0*				
Snyder et al. [4]	MVIC knee extension (kg)						
	Match 1	50.9 ± 8.0	47.9 ± 6.5	44.4 ± 7.8*			
	Match 2	44.4 ± 7.8	47.4 ± 8.1				
	MVIC knee flexion (kg)						
	Match 1	43.5 ± 4.1	38.6 ± 6.8	43.4 ± 5.2			
	Match 2	43.4 ± 5.2	37.6 ± 6.3				
	CMJ height (cm)						
	Match 1	45.6 ± 6.9	44.0 ± 6.3	43.3 ± 6.0*			
	Match 2	43.3 ± 6.0	43.3 ± 5.9				

CMJ= Countermovement jump; MVIC= maximum voluntary isometric contraction; SJ= squat jump; YoYoIE1= Yo−Yo intermittent endurance level 1; and YoYoIE2= Yo−Yo intermittent endurance level 2. *means significantly different from pre; # means significantly different from pre (time main effect). Significant differences were reported by the authors in the original studies. Missing values are data that could not be extracted by software and for which we had no response from the contacted authors. Values are as mean ± standard deviation

**Table 3:** Fatigue and recovery time course of physiological parameters

Physiological parameters							
Study	Variable	Pre	Post	1–12 h	13–24 h	25–48 h	49–72 h
	*Muscle damage markers*						
Andersson et al. [1]	CK (U.L^−1^)						
	Match 1—Active group	158 ± 136	344 ± 169*		Missing	Missing	211 ± 128
	Match 1—Passive group	146 ± 103	327 ± 214*		Missing	Missing	157 ± 120
	Match 2—Active group	211 ± 128	414 ± 161				
	Match 2—Passive group	157 ± 120	363 ± 195				
Bonilla et al. [36]	CK (U/L)	172 ± 54			169 ± 46		
	LDH (U/L)	282 ± 44			341 ± 78*		
Gravina et al. [42]	CK (U/L)	150 ± 65	243 ± 148		332 ± 302*		
	LDH (U/L)	317 ± 39	407 ± 68*		342 ± 43		
Póvoas et al. [50]	CK (U/L)						
	Match 1—High-rank players	230.8 ± 74.2	324.3 ± 124.7				
	Match 1—Low-rank players	366.4 ± 319.3	563.0 ± 356.4				
Souglis et al. [52]	CK (U/L)	145 ± 22	262 ± 55*		747 ± 290*	343 ± 185*	
Souglis et al. [2]	CK (U/L)						
	Attackers	145.70 ± 17.99	254.50 ± 15.45		494.90 ± 84.51*	422.20 ± 62.51*	340.00 ± 49.12*
	Midfielders	146.50 ± 26.26	271.40 ± 24.20		539.10 ± 65.90*	443.00 ± 62.39*	363.80 ± 53.60*
	Defenders	132.30 ± 11.47	229.40 ± 23.74		407.80 ± 24.25*	353.60 ± 25.82*	309.20 ± 18.63*
	LDH (IU/L)						
	Attackers	155.90 ± 11.44	222.30 ± 13.60		332.00 ± 18.59*	313.80 ± 16.05*	246.10 ± 11.52*
	Midfielders	152.40 ± 9.41	240.40 ± 14.40		355.50 ± 17.13	322.80 ± 11.77*	260.50 ± 14.06*
	Defenders	155.50 ± 11.07	214.80 ± 9.50		321.00 ± 13.93*	307.70 ± 14.63*	231.60 ± 10.78*
Tsubakihara et al. [53]	CK (IU/L)	210.3 ± 128.2	292.8 ± 152.2*				
	LDH (IU/L)	204.1 ± 47.5	261.9 ± 57.7*				
	*Inflammatory and immunological parameters*						
Andersson et al. [35]	IL-6 (pg/mL)						
	Match 1—Active group	2.1 ± 1.8	11.3 ± 8.3*		6.0 ± 6.9	3.5 ± 2.0	3.9 ± 4.2
	Match 1—Passive group	5.4 ± 3.4	9.8 ± 3.4*		6.1 ± 4.7	4.2 ± 1.8	4.3 ± 5.6
	Match 2—Active group	3.9 ± 4.2	10.6 ± 6.3				
	Match 2—Passive group	4.3 ± 5.6	10.1 ± 6.9				
	TNF-α (pg/mL)						
	Match 1—Active group	7 ± 2	24 ± 22*		17 ± 9	8 ± 2	10 ± 4
	Match 1—Passive group	9 ± 2	25 ± 7*		14 ± 7	8 ± 2	15 ± 11
	Match 2—Active group	10 ± 4	9 ± 2				
	Match 2—Passive group	15 ± 11	17 ± 4				
	Leukocytes (cells/mL)						
	Match 1	5.3 ± 1.1 × 10^3^	8.2 ± 1.5 × 10^3^*				4.8 ± 0.9 × 10^3^
	Match 2	4.8 ± 0.9 × 10^3^	8.3 ± 1.8 × 10^3^ *				
	Neutrophils (cells/mL)						
	Match 1	3.0 ± 1.1 × 10^3^	6.1 ± 1.5 × 10^3^*				2.7 ± 0.6 × 10^3^
	Match 2	2.7 ± 0.6 × 10^3^	6.4 ± 1.7 × 10^3^*				
	Lymphocytes (cells/mL)						
	Match 1	1.8 ± 0.3 × 10^3^	1.6 ± 0.4 × 10^3^				1.6 ± 0.4 × 10^3^
	Match 2	1.6 ± 0.4 × 10^3^	1.5 ± 0.1 × 10^3^				
Gravina et al. [42]	CRP (mg/dL)	0.06 ± 0.05	0.11 ± 0.04		0.16 ± 0.13*		
	Leukocyte (10^3^/µL)	5.35 ± 0.8	9.64 ± 2.9*		5.81 ± 1.2		
	Neutrophils (10^3^/µL)	2.54 ± 0.6	7.35 ± 2.9*		2.83 ± 0.8		
	Lymphocytes (10^3^/µL)	2.15 ± 0.5	1.62 ± 0.9*		2.29 ± 0.6		
Goulart et al. [32]	CRP (mg/L)	0.24 ± 0.19			0.73 ± 0.90	0.53 ± 0.57	0.38 ± 0.33
Póvoas et al. [50]	CRP (mg/L)						
	Match 1—High-rank players	0.75 ± 4.46	1.28 ± 1.08				
	Match 1—Low-rank players	0.96 ± 1.03	0.78 ± 0.64				
Souglis et al. [52]	CRP (mg/mL)	1.37 ± 1.31	1.53 ± 1.12		3.03 ± 2.08*	1.78 ± 1.33	
	IL-6 (pg/mL)	1.23 ± 0.71	5.09 ± 3.07*		1.35 ± 0.88	1.18 ± 0.68	
	TNF-α (pg/mL)	1.89 ± 0.53	4.49 ± 1.27*		2.18 ± 0.59	1.86 ± 0.60	
Souglis et al. [2]	CRP (mg/L)						
	Attackers	0.93 ± 0.26	1.13 ± 0.27		2.47 ± 0.59*	1.16 ± 0.41	1.03 ± 0.34
	Midfielders	0.91 ± 0.22	1.24 ± 0.31		2.88 ± 1.00*	1.12 ± 0.49	0.99 ± 0.33
	Defenders	0.99 ± 0.24	1.13 ± 0.29		2.27 ± 0.39*	1.12 ± 0.29	1.04 ± 0.26
	IL-6 (pg/mL)						
	Attackers	1.16 ± 0.15	3.54 ± 0.25*	1.16 ± 0.15		1.16 ± 0.12	1.16 ± 0.10
	Midfielders	1.15 ± 0.16	3.82 ± 0.22*	1.16 ± 0.17		1.16 ± 0.07	1.16 ± 0.13
	Defenders	1.16 ± 0.18	3.32 ± 0.22*	1.14 ± 0.18		1.17 ± 0.14	1.17 ± 0.12
Tsubakihara et al. [53]	Leukocyte (/mL)	5439 ± 1515	9497 ± 2621*				
	Neutrophil (/mL)	2915 ± 1285	7335 ± 2494*				
	Lymphocyte (/mL)	2009 ± 446	1631 ± 558*				
	*Mediators of neuroendocrine regulation*						
Broodryk et al. [37]	Cortisol (nmoll/L)						
	Match 1	9.79 ± 3.37	52.65 ± 21.43*				
	Match 2	16.22 ± 7.24	36.12 ± 16.07*				
	Match 3	22.95 ± 7.96	30.00 ± 18.97				
	Match 4	12.09 ± 3.21	29.69 ± 20.51				
	Match 5	7.04 ± 3.67	36.73 ± 10.10*				
	Match 6	6.73 ± 4.90	26.93 ± 15.00*				
Casanova et al. [3]	Cortisol (mg/dL)						
	Match 1—PIP	0.40 ± 0.13	0.42 ± 0.09				
	Match 1—GIP	0.55 ± 0.16	0.64 ± 0.48				
	Match 2—PIP	0.50 ± 0.13	0.38 ± 0.12*				
	Match 2—GIP	0.41 ± 0.11	0.49 ± 0.17				
	Match 3—PIP	0.45 ± 0.19	0.45 ± 0.12				
	Match 3—GIP	0.42 ± 0.09	0.33 ± 0.01				
	Match 4—PIP	0.73 ± 0.18	0.55 ± 0.22				
	Match 4—GIP	0.86 ± 0.20	0.60 ± 0.22				
	Testosterone (pg/mL)						
	Match 1—PIP	51.17 ± 30.52	43.50 ± 21.44				
	Match 1—GIP	74.71 ± 29.53	54.71 ± 31.23*				
	Match 2—PIP	83.63 ± 38.78	63.14 ± 14.77				
	Match 2—GIP	77.60 ± 18.05	61.40 ± 18.60				
	Match 3—PIP	68.78 ± 39.86	45.25 ± 22.33*				
	Match 3—GIP	72.33 ± 34.27	46.00 ± 14.80				
	Match 4—PIP	64.75 ± 35.08	41.25 ± 18.71				
	Match 4—GIP	59.63 ± 23.63	47.14 ± 22.79				
	T/C						
	Match 1—PIP	118.76 ± 66.03	101.26 ± 30.34				
	Match 1—GIP	136.86 ± 33.08	93.42 ± 23.39*				
	Match 2—PIP	170.32 ± 69.79	155.62 ± 40.92				
	Match 2—GIP	193.08 ± 33.71	133.22 ± 48.77				
	Match 3—PIP	164.43 ± 73.79	102.86 ± 58.34*				
	Match 3—GIP	176.55 ± 97.79	138.32 ± 45.95				
	Match 4—PIP	84.45 ± 32.84	79.17 ± 27.53				
	Match 4—GIP	71.14 ± 26.04	71.28 ± 28.27				
Casanova et al. [38]	Cortisol (mg/dL)						
	Match 1	0.48 ± 0.15	0.46 ± 0.12				
	Match 2	0.44 ± 0.12	0.39 ± 0.14				
	Match 3	0.44 ± 0.15	0.44 ± 0.15				
	Match 4	0.83 ± 0.20	0.61 ± 0.22*				
	Testosterone (pg/mL)						
	Match 1	63.0 ± 29.4	50.30 ± 24.9*				
	Match 2	78.1 ± 29.1	57.2 ± 21.1*				
	Match 3	67.6 ± 34.5	48.8 ± 22.1*				
	Match 4	63.5 ± 28.7	45.6 ± 19.5*				
Casto et al. [39]	Cortisol (μg/dL)						
	Home-loss, no OC use	0.47 ± 0.20	0.55 ± 0.21				
	Home-loss, OC use	0.33 ± 0.12	0.77 ± 0.34				
	Away-win, no OC use	0.43 ± 0.30	0.59 ± 0.25				
	Away-win, OC use	0.33 ± 0.10	0.69 ± 0.22				
	Testosterone (pg/mL)						
	Home-loss, no OC use	60.2 ± 15.1	81.2 ± 17.8				
	Home-loss, OC use	49.2 ± 10.3	72.7 ± 24.2				
	Away-win, no OC use	58.3 ± 20.4	93.0 ± 21.1				
	Away-win, OC use	48.5 ± 9.1	71.6 ± 20.6				
	Estradiol (pg/mL)						
	Home-loss, no OC use	3.71 ± 0.89	3.68 ± 0.70				
	Home-loss, OC use	3.92 ± 0.95	4.03 ± 1.10				
	Away-win, no OC use	3.27 ± 1.05	3.72 ± 1.00				
	Away-win, OC use	3.66 ± 0.75	3.86 ± 0.91				
Casto et al. [40]	Cortisol (µg/dL)						
	Win	0.36 ± 0.18	0.66 ± 0.23				
	Loss	0.38 ± 0.16	0.69 ± 0.31				
	Testosterone (pg/mL)						
	Win	51.8 ± 13.9	78.7 ± 22.5				
	Loss	53.1 ± 12.9	75.7 ± 22.0				
Edwards [41]	Cortisol (ug/dL)						
	Winning game	0.239 ± 0.143	0.485 ± 0.234*				
	Losing game	0.196 ± 0.076	0.451 ± 0.172*				
	Testosterone (pg/mL)						
	Winning game	18.40 ± 6.14	24.26 ± 7.77*				
	Losing game	17.02 ± 5.36	23.46 ± 8.66*				
Gravina et al. [42]	Testosterone (ng/mL)	0.57 ± 0.2	0.77 ± 0.3		0.56 ± 0.2		
	Estradiol (ng/mL)	68.8 ± 59	116 ± 102*		101 ± 86		
Haneishi et al. [43]	Cortisol (nmol/L)						
	Starters	18.0 ± 10.3	53.1 ± 33.9*				
	Nonstarters	12.5 ± 13.6	28.8 ± 32.5*				
Maya et al. [5]	Cortisol (nmol/L)						
	Match 1	10.18 ± 1.54	22.07 ± 7.03*				12.24 ± 4.49
	Match 2	12.24 ± 4.49	16.49 ± 7.70				
	Testosterone (nmol/L)						
	Match 1	0.27 ± 0.07	0.39 ± 0.14*				0.25 ± 0.36
	Match 2	0.25 ± 0.36	0.36 ± 0.16*				
	T/C ratio						
	Match 1	0.22 ± 0.08	0.15 ± 0.05*				0.17 ± 0.07
	Match 2	0.17 ± 0.07	0.19 ± 0.08				
Oliveira et al. [48]	Cortisol (ng/mL)						
	Winners	2.55 ± 0.53	3.12 ± 0.82				
	Losers	2.35 ± 0.23	3.00 ± 0.44				
	Testosterone (pg/mL)						
	Winners	48.2 ± 10.8	92.2 ± 15.9*				
	Losers	41.4 ± 6.5	23.4 ± 1.8 *				

CK= Creatine kinase; CRP= C-reactive protein; GIP= good individual performance; LDH= lactate dehydrogenase; OC= oral contraceptives; PIP= poor individual performance; T/C= testosterone/cortisol; and *means significantly different from pre. Significant differences were reported by the authors in the original studies. Missing values are data that could not be extracted by software and for which we had no response from the contacted authors. Values are as mean ± standard deviation

**Table 4:** Fatigue and recovery time course of perceptual parameters

Perceptual parameters							
Study	Variable	Pre	Post	1–12 h	13–24 h	25–48 h	49–72 h
Andersson et al. [1]	DOMS						
	Match 1—Active group	2.8 ± 0.8	4.3 ± 0.4*	Missing	Missing	Missing	3.5 ± 0.4
	Match 1—Passive group	3.1 ± 0.8	3.9 ± 0.8*	Missing	Missing	Missing	3.1 ± 0.8
	Match 2—Active group	3.5 ± 0.4	4.8 ± 0.6				
	Match 2—Passive group	3.1 ± 0.8	4.6 ± 0.6				
Bonilla et al. [36]	DOMS	3.2 ± 1.7	4.9 ± 1.4*		5.1 ± 1.7*		
Broodryk et al. [37]	Vigor						
	Match 1	3.8 ± 0.7	3.0 ± 0.9				
	Match 2	2.9 ± 0.8	3.3 ± 0.7				
	Match 3	3.3 ± 0.8	2.8 ± 0.9				
	Match 4	2.6 ± 0.9	3.4 ± 0.9				
	Match 5	3.4 ± 0.5	1.5 ± 0.8				
	Match 6	2.4 ± 0.8	2.6 ± 1.4				
	Fatigue						
	Match 1	1.5 ± 0.76	2.8 ± 1.2				
	Match 2	2.4 ± 1.3	2.5 ± 1.1				
	Match 3	3.1 ± 1.6	1.9 ± 1.3				
	Match 4	2.4 ± 1.7	1.6 ± 0.9				
	Match 5	2.4 ± 1.3	4.2 ± 1.0				
	Match 6	3.9 ± 0.7	2.5 ± 1.5				
Goulart et al. [32]	DOMS	0.9 ± 1.7			2.9 ± 2.5*	1.6 ± 1.8	0.7 ± 1.2
Hassmen et al. [44]	Vigor						
	Games won	18.5 ± 4.2	15.0 ± 3.4	14.8 ± 4.8			
	Games tied	20.4 ± 2.4	9.8 ± 4.2	13.3 ± 4.8			
	Games lost	17.9 ± 5.4	7.9 ± 5.1	11.7 ± 7.0			
	Fatigue						
	Games won	1.7 ± 0.8	12.8 ± 4.2	8.2 ± 4.5			
	Games tied	1.8 ± 1.0	14.0 ± 2.4	8.3 ± 3.0			
	Games lost	2.1 ± 0.9	11.2 ± 5.1	7.6 ± 4.7			
Hughes et al. [46]	DOMS	0.85 ± 0.97	5.33 ± 2.52*				
Scott et al. [51]	Soreness	1.32 ± 0.78		-0.74 ± 0.85	0.08 ± 0.88		
	Fatigue	1.50 ± 0.62		-0.82 ± 0.97	0.20 ± 0.90		

DOMS= delayed onset muscle soreness. Data from Scott et al. [51] were not included in the meta-analyses since fatigue and soreness were reported as estimated marginal means. *means significantly different from pre. Significant differences were reported by the authors in the original studies. Missing values are data that could not be extracted by software and for which we had no response from the contacted authors. Values are as mean ± standard deviation

Heterogeneity results for all parameters included in the meta-analyses are reported in the table included as Additional file 1. Missing data existed for some parameters at specific time points [1].

### Physical Performance Parameters

#### Countermovement Jump Performance

**Fig. 2:**
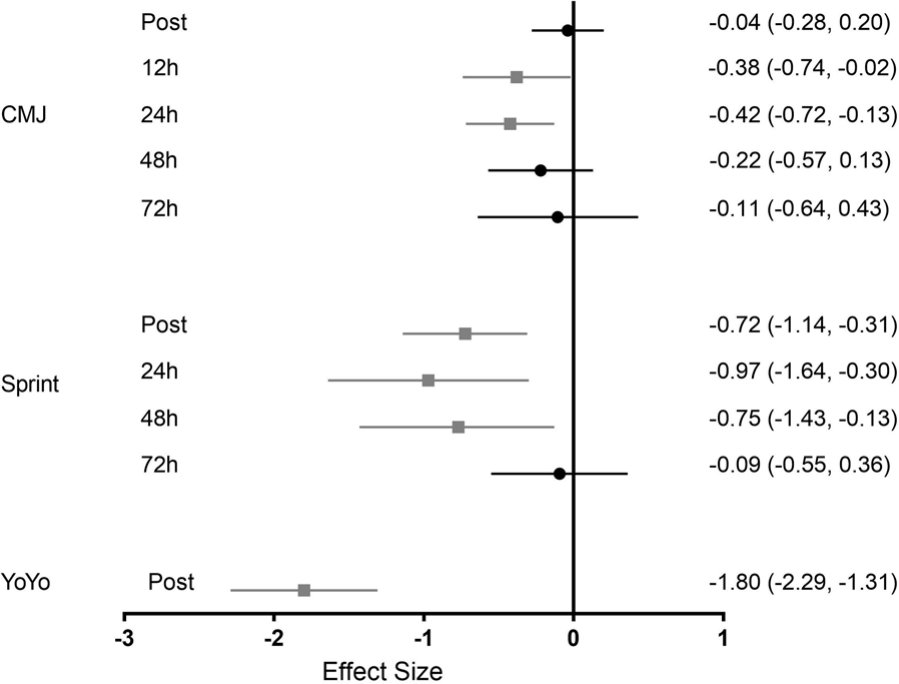
Effect size for analyses comparing post-match to pre-match physical performance parameters. Values are effect size (ES) and 95% confidence interval (CI). CMJ =  Countermovement jump; YoYo = YoYo intermittent endurance test. Black circles represent no significant difference from pre. Gray squares represent significantly different from pre

After pooling data from 13 trials (5 studies, *n* = 130 observations), a negligible and non-significant effect was observed for CMJ height immediately post-match (ES = − 0.04, 95% CI − 0.28 to 0.20). However, data from 6 trials (3 studies, *n* = 61) and data from 9 trials (4 studies, n = 93) showed CMJ height was significantly reduced with a small effect at 12 h (ES = − 0.38, 95% CI − 0.74 to − 0.02) and at 24 h post-match (ES = − 0.42, 95% CI − 0.72 to − 0.13), respectively. Further, data from 6 trials (3 studies, *n* = 63) showed a small and non-significant effect at 48 h post-match (ES = − 0.22, 95% CI − 0.57 to 0.13), suggesting full recovery at this time point for CMJ performance (Fig. 2). Finally, data from 3 trials (2 studies, *n* = 27) showed a negligible and non-significant effect at 72 h (ES = − 0.11, 95% CI − 0.64 to 0.43).

### Sprint Performance

Sprint time from 10, 20, and 30 m efforts was analyzed collectively. After pooling the data from five trials (2 studies, *n* = 48), sprint performance was significantly reduced immediately post-match with a moderate effect (ES = − 0.72, 95% CI − 1.14 to − 0.31). Data of only two trials (1 study, *n* = 20) showed sprint performance was significantly reduced with large effect at 24 h (ES = − 0.97, 95% CI − 1.64 to − 0.30) and moderate effect at 48 h (ES = − 0.75, 95% CI − 1.43 to − 0.13) post-match, though caution should be taken due to small sample size. Finally, data from four trials (2 studies, *n* = 37) showed a negligible and non-significant effect at 72 h (ES = − 0.09, 95% CI − 0.55 to 0.36), suggesting that sprint performance is recovered at this time point (Fig. 2).

### YoYo Test Performance

YoYoIE1 (1 study, *n* = 14) and YoYoIE2 (2 studies, *n* = 34) were jointly analyzed. After pooling data from three trials (3 studies, *n* = 48), the mean effect size was − 1.80 (95% CI − 2.29 to − 1.31), indicating female soccer matches induced a large and significant effect on reducing intermittent endurance capacity immediately post-match (Fig. 2).

### Physiological Parameters

#### Muscle Damage Markers

**Fig. 3:**
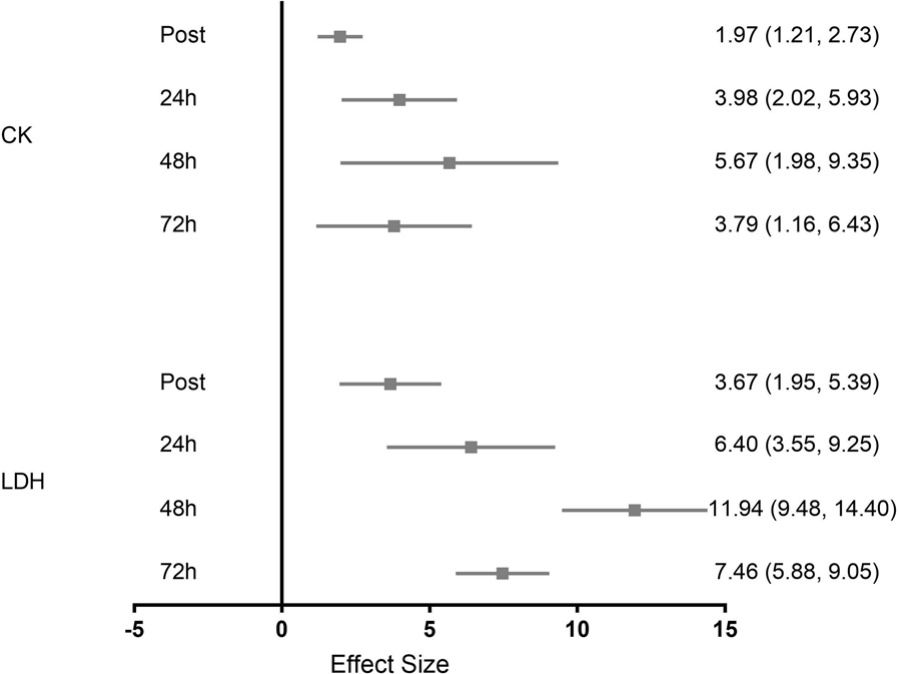
Effect size for analyses comparing post-match to pre-match muscle damage markers. Values are effect size (ES) and 95% confidence interval (CI). CK = creatine kinase; LDH = lactate dehydrogenase. Gray squares represent significantly different from pre

Blood creatine kinase and lactate dehydrogenase concentrations presented a similar time course of recovery in female soccer players. Overall, both CK and LDH were significantly increased with large effects immediately post (ES = 1.97, 95% CI 1.21–2.73; ES = 3.67, 95% CI 1.95–5.39), at 24 h (ES = 3.98, 95% CI 2.02–5.93; ES = 6.40, 95% CI 3.55–9.25), at 48 h (ES = 5.67, 95% CI 1.98–9.35; ES = 11.94, 95% CI 9.48–14.40), and still at 72 h post-match (ES = 3.79, 95% CI 1.16–6.43; ES = 7.46, 95% CI 5.88 to 9.05), respectively (Fig. 3). Importantly, these results were obtained after pooling data from 12 trials immediately post (6 studies, *n* = 144), eight trials at 24 h (5 studies, *n* = 110), six trials at 48 h (3 studies, *n* = 68), and five trials at 72 h post-match (2 studies, *n* = 47) for CK, while data from five trials immediately post (3 studies, *n* = 76) and five trials at 24 h (3 studies, *n* = 72) were used for LDH. Of note, only one study (3 trials, *n* = 30) investigated LDH responses at 48 and 72 h post-match.

### Inflammatory and Immunological Parameters

After pooling data from seven trials (4 studies, *n* = 92), a moderate and significant effect was observed for blood CRP concentration immediately post-match (ES = 0.58, 95% CI 0.28–0.88), while data from six trials (4 studies, *n* = 89) showed a significant and large effect at 24 h post-match (ES = 1.84, 95% CI 0.99–2.69). Data from five trials (3 studies, *n* = 61) showed CRP was still increased with a small effect at 48 h (ES = 0.48, 95% CI 0.12 to 0.84), while data from four trials (2 studies, *n* = 40) showed a small and non-significant effect at 72 h (ES = 0.32, 95% CI − 0.12 to 0.76), suggesting CRP returned to baseline at this time point.

**Fig. 4:**
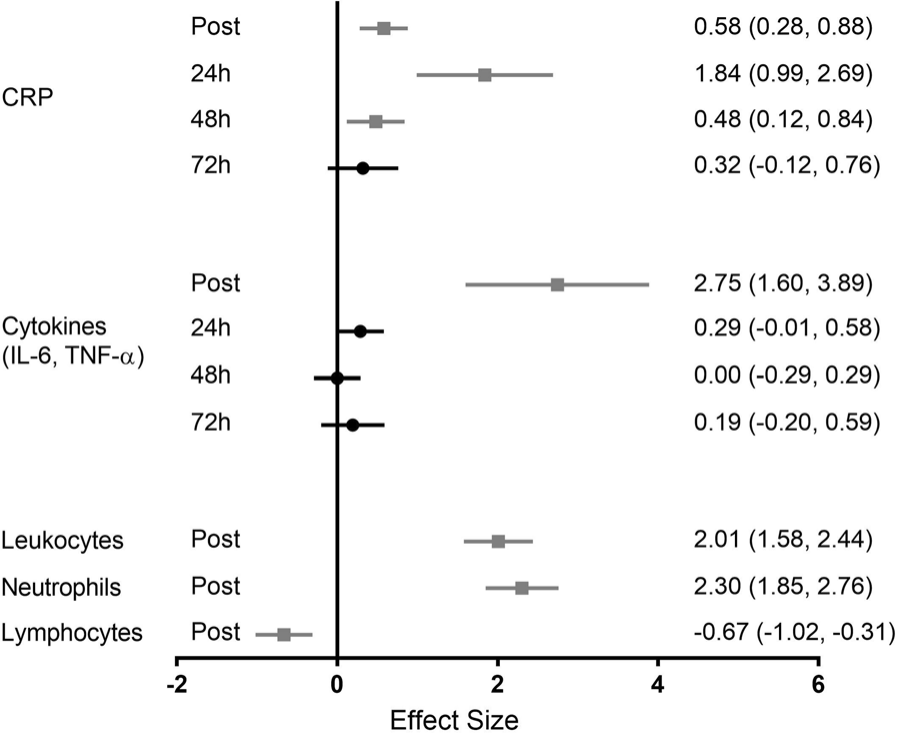
Effect size for analyses comparing post-match to pre-match inflammatory parameters. Values are effect size (ES) and 95% confidence interval (CI). CRP = C-reactive protein. Black circles represent no significant difference from pre. Gray squares represent significantly different from pre

Blood IL-6 and TNF-α concentrations were jointly analyzed. After pooling data from 13 trials (3 studies, *n* = 112), a large and significant effect was observed for increased cytokines only immediately post-match (ES = 2.75, 95% CI 1.60–3.89). Data from nine trials (3 studies, *n* = 92) showed a small and non-significant effect at 24 h (ES = 0.29, 95% CI − 0.01 to 0.58) and a negligible and non-significant effect at 48 h (ES = 0.00, 95% CI − 0.29 to 0.29]). Further, data from seven trials (2 studies, *n* = 50) also showed a negligible and non-significant effect at 72 h (ES = 0.19, 95% CI − 0.20 to 0.59) (Fig. 4).

Immunological cells were only analyzed immediately post-match. After pooling data from 4 trials (3 studies, *n* = 66), leukocytes and neutrophils were significantly increased with large effects (ES = 2.01, 95% CI 1.58–2.44 and ES = 2.30, 95% CI 1.85–2.76, respectively), while lymphocytes were significantly reduced with a large effect (ES = − 0.67, 95% CI − 1.02 to − 0.31) (Fig. 4).

### Endocrine Parameters

**Fig. 5:**
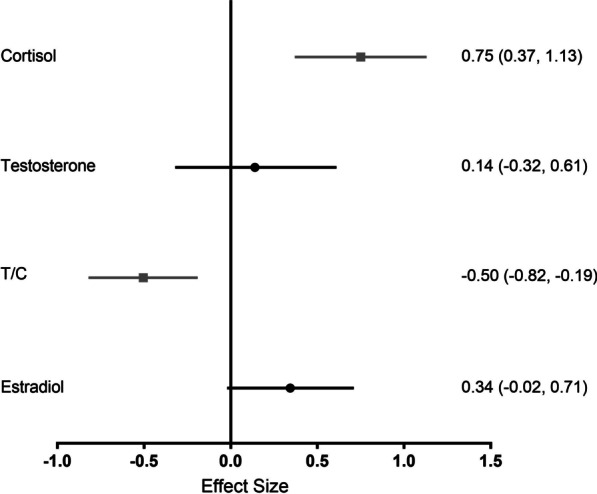
Effect size for analyses comparing immediately post-match to pre-match neuroendocrine parameters. Values are effect size (ES) and 95% confidence interval (CI). T/C = testosterone/cortisol ratio. Black circles represent no significant difference from pre. Gray squares represent significantly different from pre

Hormonal responses were the most investigated parameters in female soccer athletes, with nine studies [3, 5, 37–41, 43, 48] examining salivary samples and one study utilizing blood samples [42]. Of note, 2/10 studies evaluated free testosterone [38, 42], while the others did not specify if free or total hormone concentrations were measured. After pooling the data from 32 trials (9 studies, *n* = 334), a significant and moderate effect (ES = 0.75, 95% CI 0.37 to 1.13) was observed for increased cortisol concentration immediately post-match. Data from 25 trials (8 studies, *n* = 296) presented a negligible and non-significant effect for testosterone at post-match (ES = 0.14, 95% CI − 0.32 to 0.61) compared to pre-match. Further, after pooling data from ten trials (2 studies, *n* = 84), the testosterone/cortisol ratio was significantly decreased with a moderate effect at post-match (ES =− 0.50, 95% CI − 0.82 to − 0.19). Finally, data from five trials (2 studies, *n* = 60) showed a small and non-significant effect for salivary estradiol (ES = 0.34, 95% CI − 0.02 to 0.71) at post-match (Fig. 5).

### Perceptual Parameters

Limited data on post-match perceptual responses exist in the literature for female soccer players. After pooling, respectively, the data from six trials (3 studies, *n* = 60) and two trials (2 studies, *n* = 24), delayed onset muscle soreness was significantly increased with large effects immediately post (ES = 1.63, 95% CI 1.20–2.07) and at 24 h post-match (ES = 1.00, 95% CI 0.40–1.61)]. Only one study reported DOMS at 48 h post-match for female soccer athletes [32]. A small and non-significant effect was observed for DOMS at 72 h (ES = 0.29, 95% CI − 0.16 to 0.75) compared to pre-match (3 studies, 4 trials, *n* = 39).

**Fig. 6:**
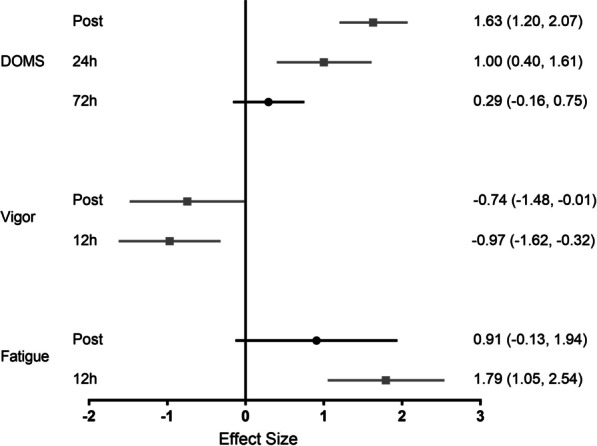
Effect size for analyses comparing post-match to pre-match perceptual parameters. Values are effect size (ES) and 95% confidence interval (CI). DOMS = delayed onset muscle soreness. Black circles represent no significant difference from pre. Gray squares represent significantly different from pre

The Brunel Mood Scale was investigated in female soccer athletes in 2 studies. Considering the post-match recovery context, only two dimensions (i.e., fatigue and vigor) were analyzed. After pooling data from nine trials (*n* = 70), a large though non-significant effect was observed for increased fatigue (ES = 0.91, 95% CI − 0.13 to 1.94), while a significant and moderate effect for reduced vigor (ES = − 0.74, 95% CI − 1.48 to − 0.01) existed immediately post-match. Data from three trials (1 study, *n* = 22) showed significant and large effects for increased fatigue (ES = 1.79, 95% CI 1.05–2.54) and reduced vigor (ES = − 0.97, 95% CI − 1.62 to − 0.32) at 12 h post-match (Fig. 6).

### Study Quality Assessment

Results of the qualitative assessment using an adapted version of the tool from Silva et al. [6] showed a mean score of 16.6 out of 26. Overall, the majority of studies reported the study question and the main outcomes to be measured clearly. However, only 11% of studies specified ground surface, and 70% of studies did not report the use of hormonal contraceptives and menstrual cycle phase. Less than half of the recovery studies reported environmental conditions and external and internal match loads. Table 5 details the quality assessment criteria and results.

**Table 5:** Qualitative assessment tool and average methodological quality scores of the 13 criteria

	Item	Qualitative assessment
1	Was the study question or objective clearly described?	1.98 ± 0.10
2	Were the inclusion criteria stated? (e.g., players with previous injuries were excluded)	1.37 ± 0.38
3	Were the main outcomes to be measured clearly described in the introduction or methods section?	1.95 ± 0.14
4	Were the main outcomes measured using accurate procedures? (e.g., reliability was reported by means of assessment or citation)	1.30 ± 0.68
5	Were the players’ participation level (e.g., amateur), training background (e.g., years of training), training status (e.g., training hours or sessions/ matches per week) described?	1.46 ± 0.39
6	Were the anthropometric characteristics described? (e.g., body mass, height, and body fat)	1.60 ± 0.74
7	Was the season period when the match took place stated? (e.g., off-season/ pre-season/ competitive-season)	1.42 ± 0.78
8	Was the ground surface specified? (e.g., grass/ artificial turf/ synthetic surface)	0.22 ± 0.64
9	Were the environmental conditions described? (e.g., temperature and humidity)	0.72 ± 0.91
10	Were external (e.g., time motion analyses/ performance measures) and internal (e.g., RPE/ heart rate) measures of match intensity recorded?	0.85 ± 0.81
11	Was the activity undertaken during the recovery period (e.g., 12–72 h post-match) described? (studies with only pre and immediately post-match measures were scored as 2)	1.90 ± 0.29
12	Was a limitation paragraph with possible confounding factors included in the study?	1.24 ± 0.88
13	Was the use of hormonal contraceptives or menstrual cycle phase reported?	0.59 ± 0.93
	Total	16.6 ± 3.0

Values are mean and standard deviation

## Discussion

The current systematic review and meta-analyses showed that female soccer matches result in acute and residual alterations in physical performance, physiological, and perceptual responses. More specifically, physical performance parameters were altered immediately post (YoYo test and sprint), at 24 h (CMJ and sprint), and until 48 h (sprint) post-match, though all physical performance measures were recovered at 72 h post-match. The inflammatory profile also showed parameter-specific responses, with IL-6 and TNF-α altered only immediately post-match, while CRP only returned to baseline at 72 h post-match. Muscle damage markers demonstrated the longest recovery time course, as CK and LDH still remained increased at 72 h post-match. Neuroendocrine regulation was partially affected by female soccer matches, with no significant differences for testosterone and estradiol immediately post-match, despite increased cortisol and a reduced testosterone/cortisol ratio at the same time point. Finally, the limited data on perceptual responses showed DOMS was increased at 24 h post-match, while vigor was reduced and fatigue increased at 12 h post-match.

Female soccer involves explosive actions, including sprinting/acceleration or peak jump height. For example, at the elite level, match demands include total distances between 9 and 11 km [18, 57], with 1.5 km covered in high-speed running (> 13 km/h) and approximately 4.7 sprints (> 22 km/h) per player [57]. Interestingly, match-induced acute fatigue did not impair the CMJ performance immediately post-match, though moderate-to-large effects for reduced intermittent aerobic capacity and sprint performance were observed. Further, reduced peak torque flexion and extension [1] and lower strength endurance [49] immediately post-match were reported in the systematic review, though not included for meta-analysis. While acute fatigue was evident for most physical performance parameters, residual fatigue was reported only for CMJ at 12–24 h and sprint time at 48 h, even though the present data lack evidence for a complete description of the recovery time course of all physical performance measures in female soccer players. The different recovery profiles for performance parameters might be explained by the different fatigability of the skill performed, muscle mass recruited, and intermuscular coordination between respective tests. Furthermore, match load variation exists between studies (i.e., competitive demands between ages or competition levels) [18, 47], and the physical load and activity pattern might affect the parameters measured.

Comparatively, Silva et al. [6] reported CMJ, YYIR1, and strength-related capabilities are still substantially reduced at 72 h post-match, while running abilities recover at 72 h in both male and female players when outcomes were merged. Bradley et al. [22] reported male players covered more total distance and more distance at higher speed thresholds than female players, though more recent data show similar total distances covered by both sexes [25, 26]. Regardless, the intensity of the match load may differ between female and male matches, and this should be considered, alongside the extent of training exposure, when interpreting fatigue and recovery responses in female players. Further, sex differences in explosive and endurance capacities (i.e., lower values in sprints, jumps, and intermittent endurance in women) [23] should also be acknowledged and may have a role in explaining the recovery process. Previous studies show that male players with higher physical qualities have lower post-match fatigue, even with a greater internal and external match load [24]; however, the current data do not concur with this view. Thus, despite lower physical match loads compared to male players, the current review would suggest lower post-match fatigue metrics for female players.

The complexity of comparing sex differences in recovery is affected by a range of factors, including training status, physical phenotype, time of the season [58], and technical–tactical and performance quality [59], physiological and menstrual cycle effects [60]. For example, differences in the morphological composition of skeletal muscle result in greater muscle oxidative capacity in females and greater glycolytic capacity in males [60]. Furthermore, females have greater mitochondrial intrinsic respiratory rates [61] and a higher density of capillaries per unit of skeletal muscle than males. The proportional fiber-type difference between sexes also influences the contractile properties, which has been suggested to contribute to a more significant fatigue resistance of female muscle during exercise [60]. Thus, sex differences in physiological aspects may also impact the post-match recovery kinetics. Despite the effect on different parameters at different time points, it seems the physical performance recovery of female players is shorter than previously reported when sex differences are not considered. In summary, performance parameters are recovered at 72 h post-match, even though the recovery in female soccer players still lacks extensive exploration, including the interaction of the confounding factors outlined above.

Soccer matches trigger a complex cascade of events involving muscle damage, inflammation, immune responses, and tissue repair [2, 42, 53]. Cytokines are glycoproteins that have context-dependent roles in the regulation and modulation of the immune response and can be grouped according to their structure or function in inflammation, for example interleukins (IL) and tumor necrosis factor (TNF) [62, 63]. The current meta-analyses showed a transient and large increase in cytokines (IL-6 and TNF-α) immediately post-match, returning to baseline at 24 h, while CRP reached peak values at 24 h and returned to baseline 72 h post-match in female players. Silva et al. [6] reported similar peaks in the recovery time course of inflammatory and immunological parameters, though these responses persisted at 72 h post-match. Thus, the early peak for IL-6 and TNF-α might regulate the hepatic secretion of CRP, explaining the later peak (24 h) for this inflammation biomarker [2, 64], and in turn, contextualizing how to interpret these post-match responses to inform recovery practices in female players.

Neutrophils and lymphocytes are subpopulations of leukocytes, playing an essential role in immune function. Neutrophils are the first subpopulation to invade injury tissue [65] and were increased immediately post-match, while lymphocytes were reduced, and total leukocytes increased with large effects. In males, the increased number of circulating leukocytes remained substantial at 48 h [6]. The acute migration of immunological cells into areas of injured tissue occurs for initiating repair [66], and this was similar for female players, though further evidence on the extended recovery time course is lacking to allow more detailed sex comparisons. Of note, males and females show marked differences in immune response to exercise when menstrual phase and hormonal contraceptives are controlled [67], thus reinforcing the need for further evidence on menstrual cycle function and immunological responses in female soccer players.

Creatine kinase and lactate dehydrogenase are useful indirect markers of muscle damage since both are intracellular enzymes with no ability to cross the sarcoplasmic barrier [68]. Both proteins showed a peak at 24 h post-match, with large effects persisting throughout the timeline investigated until 72 h. Silva et al. [6] reported similar peak profiles, with increases in CK persisting until 72 h post-match in both males and females for merged outcomes. Thus, match loads, acceleration profiles, and impacts result in muscle damage and alteration in blood enzyme concentrations [42], as evidenced by the acute and residual presence of muscle damage markers in female players. Curiously, regarding the duration of increased muscle damage markers, Souglis et al. [2] reported LDH returned to pre-match levels on the 4th day after the match in females and on the 5th day in males, while CK had not returned to pre-match levels even five days post-match in female players. Further, females had significantly lower CK and LDH over time than males [2]. The blunted muscle damage response in females could also be due to physiological differences and lower match demands reported for this population, for example, the lower distance at higher speeds covered by females compared to males [25, 26].Whereas impairments in performance measures occurred for the first 24–48 h, the typical time course for muscle damage markers returning to baseline values is more extended for both males and females. Thus, waiting for these markers to return to pre-match values before applying a new training stimulus is not feasible in high-performance settings. It is also noteworthy that the high residual CK and LDH concentrations may be due to a time lag between what happens earlier in the muscle and later in the blood, and therefore, the damage markers could still be likely augmented in the circulation, despite muscle inflammation having already been resolved [69].

Female soccer matches may also influence neuroendocrine regulation. The current meta-analysis reported increased cortisol and reduced testosterone/cortisol ratio immediately post-match, indicating physical and psychological strain [3, 39]. Thus, match demands stimulate the hypothalamus–pituitary–adrenal (HPA) axis, leading to augmented cortisol secretion from the adrenal glands [70]. Cortisol induces free fatty acids mobilization, favoring the maintenance of blood glucose to sustain match activities [71]. Further, the sex of participants and competitive context may have a role in hormone responses. For instance, while a negligible and non-significant effect for testosterone was observed here, conflicting reports on post-match testosterone in male players were reported [6]. Finally, despite estradiol possibly having some attenuating effects on exercise-induced inflammatory responses following intense physical activity [72], only small and non-significant effects for increased estradiol at post-match were reported here.

Limited data exist for perceptual responses in female players, making it challenging to complete a time course description of these parameters. The current meta-analysis showed muscle soreness peaked immediately post-match in female players and returned to baseline at 72 h, though no data at 48 h were included for analysis, despite contrasting reports of perceptual markers remaining elevated at 72 h [6]. However, our meta-analysis showed an analogous response between perceptual and performance responses, with both recovered at 48–72 h. This observation represents a faster recovery compared to that reported by Silva et al. [6], which jointly investigated male and female responses. Furthermore, large effects for increased fatigue and reduced vigor were observed at 12 h post-match. Of note, only two studies investigated the Brunel Mood Scale during official matches in female players [37, 44], both presenting outcomes of multiple matches. Thus, interpreting such results might also include the cumulative effect of match demands during a female soccer tournament.

Overall, the results of the present systematic review and meta-analysis differed from those presented by Silva et al. [6] and showed a faster recovery for female players. However, some considerations should be acknowledged. For example, despite sex-merged data from the 42 studies included in the Silva et al. [6] meta-analysis, only 10 reported post-match recovery in females, while we included 26 studies in the meta-analysis. Thus, the previously published findings were possibly more influenced by male soccer data, explaining the differences reported between the current and Silva et al. [6] results. Furthermore, the shorter recovery time course for females may be due to lower external match loads compared to male players [22], though both studies included all playing levels. Finally, our meta-analysis focused on fatigue and recovery timeline from ecologically valid contexts (official matches), while Silva et al. [6] also included studies whose intervention consisted of on-field and laboratory simulation protocols.

Despite evidence showing the menstrual cycle influences the parameters investigated, only 30% of the studies reported the use of hormonal contraceptives or the menstrual cycle phase. This percentage is similar to those reported in a recent systemic review of the representativeness of women in thermoregulation research, in which less than 30% of articles that included women reported their menstrual orientations (e.g., natural menstruating, hormonal contraceptive user, pregnant, and postmenopausal) and only 22% reported both menstrual orientation and phase [73]. Among the studies investigating female soccer, Bonilla et al. [36] and Broodryk et al. [37] evaluated the players only during the follicular phase to avoid variations in the data due to the menstrual cycle. Controlling for the menstrual cycle is relevant considering that CRP is increased in the early follicular phase [74], while reports of higher cortisol during the luteal phase also exist [75]. Further, previous research showed premenstrual syndrome influenced the inflammatory condition, mood states, and stress hormones in female soccer players [76]. Thus, the post-match recovery time course during different menstrual cycle phases is a relevant topic for future research.

Whilst this meta-analysis reports novel findings specific to female soccer players, a noted limitation was the small number of studies investigating the post-match recovery on this population. Thus, subgroups analyses for the level of players or different match conditions (i.e., single match vs. congested schedules) were not possible. Further, the number of trials was progressively reduced over the recovery time course, making the description of residual fatigue limited. Finally, due to the small sample size, we grouped some parameters for analyses, such as the cytokines, different distances of sprint tests, and levels of YoYo tests. This grouping may hide the response of individual parameters (i.e., TNF-*α* and IL-6) that, when analyzed separately, may demonstrate a different response.

## Conclusion

Our systematic analysis reveals that most performance, physiological, and perceptual parameters in female soccer players are recovered at 48 h post-match, except for sprint performance, CRP, CK, and LDH that require at least 72 h to return to pre-match values. Such detailed recovery time course analyses can be used to provide specific planning and training information for match preparation and scheduling. Finally, during congested schedules, an interval between soccer matches longer than 48 h is recommended to ensure appropriate recovery in female players.

## Supplementary Information

**Additional file 1.** Supplementary table 1.

## Data Availability

The datasets analyzed during the current study are available from the corresponding author on reasonable request.

## Abbreviations

A: Amateur
ATT: Attackers
C: College
CB: Center back
CM: Center midfield
CMJ: Countermovement jump
CK: Creatine kinase
CRP: C-reactive protein
DEF: Defenders
DOMS: Delayed onset muscle soreness
E: Elite
F: Forward
FB: Full back
GIP: Good individual performance
HL: High level
IL: Interleukin
LDH: Lactate dehydrogenase
MID: Midfielders
MVIC: Maximum voluntary isometric contraction
OC: Oral contraceptives
PIP: Poor individual performance
R: Recreational
RH: Relative humidity
SJ: Squat jump
T/C: Testosterone/cortisol
TNF: Tumor necrosis factor
WM: Wide midfield
YoYoIE1: YoYo intermittent endurance level 1
YoYoIE2: YoYo intermittent endurance level 2
