# Supplementary material for: Fatigue and Recovery Time Course After Female Soccer Matches: A Systematic Review And Meta-analysis
Source: Sports Med Open. 2022 Jun 3;8:72. doi: 10.1186/s40798-022-00466-3 (PMC9166924; doi:10.1186/s40798-022-00466-3)
Supplement: Supplementary file 1 — Additional file 1. Supplementary table 1. [file 40798_2022_466_MOESM1_ESM.docx]

**Fatigue and recovery time course after female soccer matches: a systematic review and meta-analysis**

**Sports Medicine**

Karine Naves de Oliveira Goulart^1,2^, Cândido Celso Coimbra^3^, Helton Oliveira Campos^3,4^, Lucas Rios Drummond^3^, Pedro Henrique Madureira Ogando^1^, Georgia Brown^2^, Bruno Pena Couto^5^, Rob Duffield^2,6^, Samuel Penna Wanner^1^

^1^ Exercise Physiology Laboratory, School of Physical Education, Physiotherapy and Occupational Therapy, Universidade Federal de Minas Gerais, Belo Horizonte (MG), Brazil.

^2^ School of Sport, Exercise and Rehabilitation, Faculty of Health, University of Technology Sydney (UTS), Moore Park (NSW), Australia.

^3^ Department of Physiology and Biophysics, Institute of Biological Sciences, Universidade Federal de Minas Gerais, Belo Horizonte (MG), Brazil.

^4^ Department of Biological Sciences, Universidade do Estado de Minas Gerais – Unidade Carangola, Carangola (MG), Brazil.

^5^ School of Health and Behavioural Sciences, University of the Sunshine Coast, Sippy Downs (Qld), Australia.

^6^ Medical Department, Football Australia, Sydney, Australia

Corresponding author:

Karine Naves de Oliveira Goulart, PhD

karinegoulart91@gmail.com

Supplementary table. Measures of statistical heterogeneity for each parameter included in the meta-analysis.

| Parameters | Time-points | Effects | I_2_ (%) | Q | Df | P |
| --- | --- | --- | --- | --- | --- | --- |
| CMJ | Pre vs Post | Fixed | 0.0 | 2.19 | 12 | 0.999 |
|  | Pre vs 12 h | Fixed | 0.0 | 1.46 | 5 | 0.917 |
|  | Pre vs 24 h | Fixed | 0.0 | 3.40 | 8 | 0.907 |
|  | Pre vs 48 h | Fixed | 0.0 | 0.18 | 5 | 0.999 |
|  | Pre vs 72 h | Fixed | 0.0 | 0.45 | 2 | 0.800 |
|  |  |  |  |  |  |  |
| Sprint | Pre vs Post | Fixed | 0.0 | 0.34 | 4 | 0.987 |
|  | Pre vs 24 h | Fixed | 0.0 | 0.57 | 1 | 0.452 |
|  | Pre vs 48 h | Fixed | 0.0 | 0.39 | 1 | 0.534 |
|  | Pre vs 72 h | Fixed | 0.0 | 0.79 | 3 | 0.851 |
|  |  |  |  |  |  |  |
| YoYo | Pre vs Post | Fixed | 40.8 | 3.38 | 2 | 0.185 |
|  |  |  |  |  |  |  |
| CK | Pre vs Post | Random | 84.7 | 71.99 | 11 | 0.001 |
|  | Pre vs 24 h | Random | 94.4 | 88.57 | 5 | 0.001 |
|  | Pre vs 48 h | Random | 93.2 | 43.89 | 3 | 0.003 |
|  | Pre vs 72 h | Random | 93.5 | 61.30 | 4 | 0.001 |
|  |  |  |  |  |  |  |
| LDH | Pre vs Post | Random | 90.6 | 42.58 | 4 | 0.001 |
|  | Pre vs 24 h | Random | 95.0 | 80.31 | 4 | 0.001 |
|  | Pre vs 48 h | Fixed | 0.0 | 1.95 | 2 | 0.378 |
|  | Pre vs 72 h | Fixed | 0.0 | 0.96 | 2 | 0.619 |
|  |  |  |  |  |  |  |
| CRP | Pre vs Post | Fixed | 36.8 | 9.49 | 6 | 0.148 |
|  | Pre vs 24 h | Random | 79.7 | 24.69 | 5 | 0.001 |
|  | Pre vs 48 h | Fixed | 0.0 | 0.60 | 4 | 0.964 |
|  | Pre vs 72 h | Fixed | 0.0 | 0.25 | 3 | 0.970 |
|  |  |  |  |  |  |  |
| Cytokines | Pre vs Post | Random | 88.1 | 100.84 | 12 | 0.001 |
|  | Pre vs 24 h | Fixed | 0.0 | 5.48 | 8 | 0.705 |
|  | Pre vs 48 h | Fixed | 0.0 | 2.50 | 8 | 0.962 |
|  | Pre vs 72 h | Fixed | 0.0 | 2.50 | 6 | 0.868 |
|  |  |  |  |  |  |  |
| Leukocytes | Pre vs Post | Fixed | 0.0 | 0.46 | 3 | 0.928 |
|  |  |  |  |  |  |  |
| Lymphocytes | Pre vs Post | Fixed | 0.0 | 0.24 | 3 | 0.972 |
|  |  |  |  |  |  |  |
| Neutrophils | Pre vs Post | Fixed | 0.0 | 0.58 | 3 | 0.900 |
|  |  |  |  |  |  |  |
| Cortisol | Pre vs Post | Random | 79.8 | 153.61 | 31 | 0.001 |
|  |  |  |  |  |  |  |
| Testosterone | Pre vs Post | Random | 85.3 | 162.79 | 24 | 0.001 |
|  |  |  |  |  |  |  |
| T/C | Pre vs Post | Fixed | 34.4 | 13.73 | 9 | 0.132 |
|  |  |  |  |  |  |  |
| Estradiol | Pre vs Post | Fixed | 0.0 | 1.44 | 4 | 0.837 |
|  |  |  |  |  |  |  |
| DOMS | Pre vs Post | Fixed | 34.2 | 7.60 | 5 | 0.179 |
|  | Pre vs 24 h | Fixed | 0.0 | 0.09 | 1 | 0.763 |
|  | Pre vs 72 h | Fixed | 10.0 | 3.33 | 3 | 0.343 |
|  |  |  |  |  |  |  |
| Vigor | Pre vs Post | Random | 73.8 | 30.53 | 8 | 0.001 |
|  | Pre vs 12 h | Fixed | 0.0 | 0.66 | 2 | 0.718 |
|  |  |  |  |  |  |  |
| Fatigue | Pre vs Post | Random | 84.4 | 51.29 | 8 | 0.001 |
|  | Pre vs 12 h | Fixed | 0.0 | 0.65 | 2 | 0.722 |

^CMJ= countermovement jump; YoYo= Yo-yo intermittent endurance test; CK= Creatine kinase; LDH= Lactate Dehydrogenase; CRP= C-reactive protein; DOMS= delayed onset muscle soreness; df= degrees of freedom.^
